# Clinical Relationship between Steatocholecystitis and Gallbladder Contractility Measured by Cholescintigraphy

**DOI:** 10.1155/2015/730930

**Published:** 2015-01-29

**Authors:** Chang Seok Bang, Yong Sub Lee, Jai Hoon Yoon, Youn Jeong Kim, Jin Bong Kim, Dong Joon Kim

**Affiliations:** ^1^Department of Internal Medicine, Hallym University College of Medicine, 153 Gyo-dong, Chuncheon 200703, Republic of Korea; ^2^Department of Internal Medicine, The Catholic University of Korea College of Medicine, Seoul, Republic of Korea

## Abstract

*Objective.* Contractility of gallbladder is known to be decreased in fatty gallbladder diseases. However, clinical estimation data about this relationship is still lacking. The aim of this study was to investigate the association between steatocholecystitis and contractility of gallbladder. *Methods.* Patients with cholecystitis (steatocholecystitis versus nonsteatocholecystitis) who underwent cholescintigraphy before cholecystectomy were retrospectively evaluated in a single teaching hospital of Korea. The association of steatocholecystitis with contractility of gallbladder, measured by preoperative cholescintigraphy, was assessed by univariable and multivariable analysis. *Results.* A total of 432 patients were finally enrolled (steatocholecystitis versus nonsteatocholecystitis; 75 versus 357, calculous versus acalculous cholecystitis; 316 versus 116). In the multivariable analysis, age (OR: 0.94, 95% CI: 0.90–0.99, *P* = 0.01) and total serum cholesterol (OR: 1.02, 95% CI: 1.01–1.04, *P* = 0.04) were related to steatocholecystitis in patients with acalculous cholecystitis. Only age (OR: 0.97, 95% CI: 0.94–0.99, *P* = 0.004) was significantly related to steatocholecystitis in patients with calculous cholecystitis. However, ejection fraction of gallbladder reflecting contractility measured by cholescintigraphy was not related to steatocholecystitis irrespective of presence of gallbladder stone in patients with cholecystitis. *Conclusion.* Ejection fraction of gallbladder measured by cholescintigraphy cannot be used for the detection or confirmation of steatocholecystitis.

## 1. Introduction

With the increasing prevalence of obesity, fatty infiltrative disease in the internal organs has been noted [1, 2]. Fatty gallbladder disease includes cholesterol polyp or cholesterolosis results from abnormal fatty deposition in the gallbladder mucosa [3, 4]. Pathogenic links among insulin resistance, hyperinsulinemia, and fatty gallbladder disease have been evaluated. Patients with obesity have increased cholesterol saturation in bile, which is induced by increased cholesterol synthesis and secretion of bile cholesterol [5, 6]. Long-standing fatty deposition induces steatocholecystitis through chronic inflammation and tissue damage [7]. Recently, this type of cholecystitis is increasing and taking a substantial portion of cholecystectomy, even without definite gallbladder stone [7, 8].

Contractility of gallbladder is known to be decreased in fatty gallbladder disease [7, 9]. This is induced by abnormal wall structure and decreased response of gallbladder to the neurotransmitter associated with oxidative stress and insulin resistance [10–12]. However, clinical estimation data about the relationship between steatocholecystitis and contractility of gallbladder is still lacking. The aim of this study was to investigate the relationship between steatocholecystitis and contractility of gallbladder, using preoperative cholescintigraphy.

## 2. Materials and Methods

### 2.1. Ethics Statement

This study was conducted in accordance with the Declaration of Helsinki and approved by an institutional review board of Chuncheon Sacred Heart hospital before initiating the study (2013-85). Patient records or information was anonymized and deidentified prior to analysis.

### 2.2. Patients and Methods

From January 2007 through July 2013, a total of 454 patients who had undergone preoperative cholescintigraphy because of cholecystitis were retrospectively evaluated in a single teaching hospital of Korea. Steatocholecystitis was determined as the state of cholecystitis combined with cholesterolosis or cholesterol polyp in gallbladder according to the final pathology report. Patients with adenomyoma, adenomyomatosis, adenoma, dysplasia, or cancer of the gallbladder, which could potentially influence the contractility of gallbladder, were all excluded. The association of steatocholecystitis with contractility of gallbladder measured by cholescintigraphy was assessed by univariable and multivariable analysis.

### 2.3. Cholescintigraphy

All the enrolled patients underwent cholescintigraphy before cholecystectomy for the assessment of biliary pain or cholecystitis. Patients were fasted for at least 8 hours not longer than 12 hours before administration of technetium labeled agent. The 99m-technetium trimethylbromo-iminodiacetic acid (mebrofenin; Amersham-GE, London, UK) was used. Sequential 5, 10, 20, 30, 45, and 60 minutes anterior images of the abdomen were obtained after the intravenous administration of 100 mCi radiopharmaceutical agent. Then, the patients ingested standard high fat diet consisting of eggs and sandwiches and sequential anterior images of the abdomen were obtained after 80, 100, and 120 minutes. If the gallbladder was not visualized within 120 minutes, delayed images for up to 240 min were obtained. The interpretation of the cholescintigraphy was based on the presence or absence of biliary excretion (visualization of tracer activity in the gallbladder). If no radioactivity was detected in the gallbladder area at 4 hours after the infusion, the patient was classified as having nonvisualization of the gallbladder and the scintigraphic recording was stopped. For the calculation of gallbladder ejection fraction (GBEF), standard nuclear medicine software was used based on the following equation: GBEF (%) = (net gallbladder_max⁡_) − (net gallbladder_min⁡_) × 100/net gallbladder_max⁡_ [13]. The nonvisualization of gallbladder after 4 hours on scintigraphy was defined as zero % of GBEF.

### 2.4. Histopathology

All resected gallbladders of enrolled patients were pathologically examined. Resected gallbladders were immediately placed in a 10% neutral buffered formalin, routinely processed and embedded in paraffin en bloc. Two sections were stained with hematoxylin and eosin. All histopathologic examinations were done by clinical pathologists and the final pathologic diagnosis was established based on consensus of two expert pathologists.

### 2.5. Statistical Analysis

Student *t*-test was used for the assessment of continuous variables and data were expressed as mean ± standard deviation (SD). Fisher's exact test was used to assess the categorical variables and data were expressed as number and percentage (*n*, %). A multivariable logistic regression test was used to detect the independent risk factors related to steatocholecystitis. A *P* value <0.05 was considered significant for all tests. All the analyses were performed using the SPSS software, version 21.0 (IBM Corp., Armonk, NY, USA).

## 3. Results

### 3.1. Clinical Characteristics

**Figure 1:**
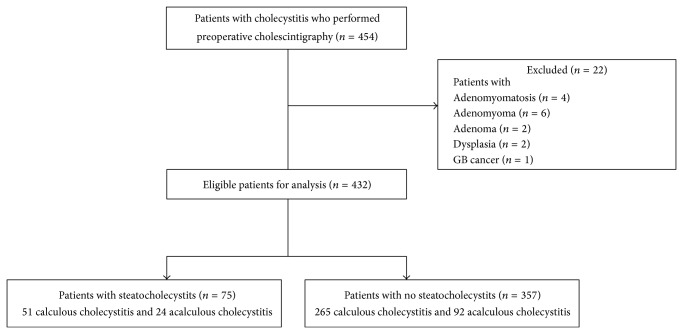
Flow chart of study design.

During the 6-year study period, 454 patients who underwent preoperative cholescintigraphy and cholecystectomy were identified. The distribution of excluded cases is as follows: adenomyomatosis (*n* = 11), adenomyoma (*n* = 6), adenoma (*n* = 2), dysplasia (*n* = 2), and gallbladder cancer (*n* = 1) (Figure 1). After exclusion, 432 eligible patients for analysis were finally enrolled. The clinical characteristics of these patients are demonstrated in Tables 1 and 2. The mean age of the patients was 54.4 ± 15.7 (mean ± SD). Two hundred two (46.8%) men and 230 (53.2%) women were enrolled. The proportion of the patients who had gallbladder stone was 73.1% (*n* = 316). The mean body mass index (BMI) of the patients was 24.9 ± 3.6 (mean ± SD). The characteristics of serum lipid profile were 168.2 ± 39.3 in total cholesterol, 110.4 ± 73 in triglyceride (TG), 98.7 ± 31.3 in low-density lipoprotein (LDL), and 50.9 ± 14.5 in high-density lipoprotein (HDL), respectively (mg/dL, mean ± SD). The mean GBEF estimated by cholescintigraphy was 41.2 ± 41.7 (%, mean ± SD). The most important variable which could influence the GBEF measured by cholescintigraphy in patients with cholecystitis is gallbladder stone. Thus, authors performed separate analyses by patients with acalculous or calculous cholecystitis. There was no statistically significant difference in the entire estimated baseline characteristic between patients with acalculous and calculous cholecystitis (Table 2).

### 3.2. Univariable Analysis of Risk Factors for Steatocholecystitis

The incidence of steatocholecystitis was 17.4% (*n* = 75) in the total population, 20.7% (*n* = 24) in patients with acalculous cholecystitis, and 16.1% (*n* = 51) in patients with calculous cholecystitis (Tables 3 and 5). In the univariable analysis for the risk factors for steatocholecystitis (versus nonsteatocholecystitis), age, sex, BMI, aspartate aminotransferase (AST), total bilirubin, total cholesterol, LDL, and GBEF were statistically different variables in patients with acalculous cholecystitis (Table 3). Meanwhile, age, sex, hypertension (HTN), BMI, and GBEF were statistically significant variables in patients with calculous cholecystitis for the development of steatocholecystitis (versus nonsteatocholecystitis) (Table 5). Age, sex, BMI, and GBEF were the commonly detected statistically significant variables. Patients with steatocholecystitis were younger, mostly women, with higher BMI and GBEF performance.

### 3.3. Multivariable Analysis of Risk Factors for Steatocholecystitis

In the multivariate analysis for clinical risk factors of steatocholecystitis, age (OR: 0.94, 95% CI: 0.90–0.99, *P* = 0.01) and total serum cholesterol (OR: 1.02, 95% CI: 1.01–1.04, *P* = 0.04) were associated with development of steatocholecystitis in patients with acalculous cholecystitis (Table 4). Meanwhile, only age (OR: 0.97, 95% CI: 0.94–0.99, *P* = 0.004) was significantly related to steatocholecystitis in patients with calculous cholecystitis (Table 6). GBEF reflecting contractility was not related to steatocholecystitis irrespective of presence of gallbladder stone in patients with cholecystitis.

## 4. Discussion

Cholesterolosis and cholesterol polyps are incidental findings detected in the cholecystectomy specimen or ultrasonic examination. Recently, however, the patients with fatty gallbladder disease are increasing and steatocholecystitis is taking a substantial portion of cholecystectomy, even without definite gallbladder stone [7, 8]. Diagnosis is usually made by ultrasound. However, there are no specific findings indicating diffuse cholesterolosis and subjective diagnosis is usually made.

In the present study, the authors evaluated the relationship between steatocholecystitis and contractility of gallbladder, using preoperative cholescintigraphy. Another outcome was to assess the feasibility of cholescintigraphy in the detection or confirmation of steatocholecystitis. Previous studies indicated that contractility of gallbladder is decreased in patients with fatty gallbladder diseases [14–17]. The mechanism was postulated as an abnormal fatty deposition in the gallbladder mucosa associated with chronic inflammation and tissue damage, which could result in deterioration of gallbladder function [3, 4, 7]. Other multiple pathogenic links that have been reported are insulin resistance, hyperglycemia, leptin hormonal dysfunction, autonomic neuropathy, and oxidative stress [10–12]. However, clinical data which estimated the relationship between steatocholecystitis and contractility of gallbladder is lacking.

Based on the results of our study, the contractility of gallbladder measured by cholescintigraphy was not associated with steatocholecystitis. This result was consistent, irrespective of presence of gallbladder stone. The discrepancy between the results of previous studies and the present data may be explained by pitfalls of cholescintigraphy. Cholescintigraphy has the highest diagnostic accuracy of all imaging modalities for the detection of acute cholecystitis [18]. However, there are several pitfalls in this examination. False-positivity in some conditions, including insufficient or prolonged fasting state, severe hepatocellular disease, bile duct obstruction, or severe chronic cholecystitis, could lead to the inaccurate diagnosis [13]. The most important drawback is that nonvisualized gallbladder is estimated at zero % of GBEF. In patients with acute cholecystitis, cystic duct stone or gallbladder edema even without obstructing stone could result in nonvisualized gallbladder in cholescintigraphy. This means an estimation of gallbladder contractility could be zero %, even in the situation of preserved contractile function. In contrast, incomplete cystic duct obstruction or mild gallbladder wall edema could lead to false negative result in the diagnosis of acute cholecystitis. Another limitation of cholescintigraphy is that a single measurement of GBEF is limited in its representativeness. The timing of the measurement could be a substantial bias. The administration of antibiotics or biliary drainage could result in a change of the contractile function measurements.

Interesting data relevant to contractility of the gallbladder was a higher trend of GBEF in patients with steatocholecystitis, although statistically insignificant after adjusting for confounding factors (Tables 3–6). This finding is opposite from the noted concept and there has been no evidence for the association between higher gallbladder contractility and steatocholecystitis. According to our study, higher GBEF was associated with steatocholecystitis, irrespective of presence of gallbladder stone in the univariable analysis (Tables 3 and 5). However, there was no significant association in the multivariate analysis. It is inferred that zero % of GBEF stated above could lead to this inverse relationship and this impact was diminished after adjusting confounding factors (Tables 4 and 6).

In contrast to the previous study performed in Greece, patients with acute cholecystitis were relatively younger in this study [19]. Moreover, patients with steatocholecystitis were younger than patients without steatocholecystitis. The mechanism or reason is unclear and further large scale studies are needed to elucidate this issue.

This study has several limitations. It is a retrospective study from a single hospital and a small number of patients were enrolled for the statistical analysis. Another limitation was the lacking information about the type and number of gallbladder stone, antibiotic treatment, and the time of measurement of GBEF. However, contrary to the previous studies, contractile function of the gallbladder was quantified and compared directly between patients with steatocholecystitis and with no steatocholecystitis in our study.

## 5. Conclusion

In conclusion, ejection fraction of gallbladder measured by cholescintigraphy cannot be used for the detection or confirmation of patients with steatocholecystitis. Future studies for the development of biomarkers or surrogate markers reflecting contractile function of gallbladder or indicating fatty gallbladder disease are needed for the diagnosis of steatocholecystitis.

## Figures and Tables

**Table 1:** Clinical characteristics of total enrolled patients.

Variable	Acute cholecystitis (*n* = 432)
Age (years, mean ± SD)	54.4 ± 15.7
Men/women (%)	202 (46.8)/230 (53.2%)
DM (%)	48 (11.1)
HTN (%)	135 (31.3)
GB stone (%)	316 (73.1)
BMI (kg/m^2^, mean ± SD)	24.9 ± 3.6
Hb (g/dL, mean ± SD)	13.6 ± 1.8
Platelet (10^3^/mm^3^, mean ± SD)	263.3 ± 78.9
AST (IU/L, mean ± SD)	30.3 ± 24.9
ALT (IU/L, mean ± SD)	35.1 ± 36.5
Total bilirubin (mg/dL, mean ± SD)	1.0 ± 3.1
Total cholesterol (mg/dL, mean ± SD)	168.2 ± 39.3
TG (mg/dL, mean ± SD)	110.4 ± 73
LDL (mg/dL, mean ± SD)	98.7 ± 31.3
HDL (mg/dL, mean ± SD)	50.9 ± 14.5
GBEF (%, mean ± SD)	41.2 ± 41.7

SD: standard deviation; *n*: number; DM: diabetes mellitus; HTN: hypertension; GB: gallbladder; BMI: body mass index; Hb: hemoglobin; AST: aspartate aminotransferase; ALT: alanine aminotransferase; TG: triglyceride; LDL: low-density lipoprotein; HDL: high-density lipoprotein; GBEF: gallbladder ejection fraction.

**Table 2:** Clinical characteristics of patients with cholecystitis.

Variable	Acalculous cholecystitis (*n* = 116)	Calculous cholecystitis (*n* = 316)	*P* value
Age (years, mean ± SD)	55.7 ± 15.1	53.9 ± 16	0.31
Men/women (%)	56 (48.3)/60 (51.7%)	146 (46.2)/170 (53.8)	0.75
DM (%)	11 (9.5)	37 (11.7)	0.61
HTN (%)	36 (31)	99 (31.3)	>0.99
BMI (kg/m^2^, mean ± SD)	24.6 ± 3.4	25 ± 3.7	0.25
Hb (g/dL, mean ± SD)	13.6 ± 1.7	13.6 ± 1.9	0.99
Platelet (10^3^/mm^3^, mean ± SD)	271.1 ± 81.1	260.4 ± 78	0.21
AST (IU/L, mean ± SD)	30.9 ± 18.1	30.1 ± 27	0.78
ALT (IU/L, mean ± SD)	34.1 ± 27.6	35.4 ± 39.3	0.74
Total bilirubin (mg/dL, mean ± SD)	0.9 ± 0.8	1.1 ± 3.6	0.60
Total cholesterol (mg/dL, mean ± SD)	169.2 ± 40.2	167.9 ± 38.9	0.76
TG (mg/dL, mean ± SD)	109.6 ± 80.6	110.9 ± 70.1	0.88
LDL (mg/dL, mean ± SD)	98.3 ± 30.5	98.8 ± 31.7	0.90
HDL (mg/dL, mean ± SD)	50.6 ± 12.6	51 ± 15.1	0.84
GBEF (%, mean ± SD)	38.4 ± 40.8	42.3 ± 42	0.39

SD: standard deviation; *n*: number; DM: diabetes mellitus; HTN: hypertension; GB: gallbladder; BMI: body mass index; Hb: hemoglobin; AST: aspartate aminotransferase; ALT: alanine aminotransferase; TG: triglyceride; LDL: low-density lipoprotein; HDL: high-density lipoprotein; GBEF: gallbladder ejection fraction.

**Table 3:** Univariable analysis for clinical risk factors of steatocholecystitis in patients with acalculous cholecystitis.

Variable	Steatocholecystitis (*n* = 24, 20.7%)	Nonsteatocholecystitis (*n* = 92, 79.3%)	*P* value
Age (years, mean ± SD)	46.8 ± 10.8	58 ± 15.2	<0.001
Men/women (%)	7 (29.2)/17 (70.8)	49 (53.3)/43 (46.7)	0.04
DM (%)	1 (4.2)	10 (10.9)	0.46
HTN (%)	7 (29.2)	29 (31.5)	>0.99
BMI (kg/m^2^, mean ± SD)	26.0 ± 3.8	24.2 ± 3.2	0.02
Hb (g/dL, mean ± SD)	13.5 ± 1.2	13.6 ± 1.8	0.76
Platelet (10^3^/mm^3^, mean ± SD)	284.7 ± 69.1	267.6 ± 83.9	0.36
AST (IU/L, mean ± SD)	25.6 ± 10.3	32.3 ± 19.5	0.03
ALT (IU/L, mean ± SD)	31.8 ± 28.6	34.7 ± 27.4	0.65
Total bilirubin (mg/dL, mean ± SD)	0.7 ± 0.3	1.0 ± 0.9	0.01
Total cholesterol (mg/dL, mean ± SD)	188.3 ± 37.7	164.4 ± 39.6	0.01
TG (mg/dL, mean ± SD) (median/IQR)	102.1 ± 65 76/69–120	111.4 ± 84.4 91.5/59.5–126.8	0.66 0.65
LDL (mg/dL, mean ± SD)	113.1 ± 25.1	94.6 ± 30.8	0.02
HDL (mg/dL, mean ± SD)	51.7 ± 10	50.3 ± 13.2	0.67
GBEF (%, mean ± SD)	61 ± 38.1	32.5 ± 39.6	0.002

SD: standard deviation; *n*: number; DM: diabetes mellitus; HTN: hypertension; GB: gallbladder; BMI: body mass index; Hb: hemoglobin; AST: aspartate aminotransferase; ALT: alanine aminotransferase; TG: triglyceride; IQR: interquartile range; LDL: low-density lipoprotein; HDL: high-density lipoprotein; GBEF: gallbladder ejection fraction.

**Table 4:** Multivariable analysis for clinical risk factors of steatocholecystitis in patients with acalculous cholecystitis.

Variables for steatocholecystitis	OR (95% CI)	*P* value
Age	0.94 (0.90–0.99)	0.01
Sex	NS	NS
BMI	1.19 (0.99–1.42)	0.06
AST	NS	NS
Total bilirubin	NS	NS
Total cholesterol	1.02 (1.01–1.04)	0.04
LDL	NS	NS
GBEF	1.03 (0.99–1.03)	0.08

OR: odds ratio; CI: confidence interval; NS: not significant; BMI: body mass index; AST: aspartate aminotransferase; LDL: low-density lipoprotein; GBEF: gallbladder ejection fraction.

**Table 5:** Univariable analysis for clinical risk factors of steatocholecystitis in patients with calculous cholecystitis.

Variable	Steatocholecystitis (*n* = 51, 16.1%)	Nonsteatocholecystitis (*n* = 265, 83.9%)	*P* value
Age (years, mean ± SD)	46.2 ± 12.6	55.4 ± 16.1	<0.001
Men/women (%)	13 (25.5)/38 (74.5)	133 (50.2)/132 (49.8)	0.001
DM (%)	5 (9.8)	32 (12.1)	0.81
HTN (%)	9 (17.6)	90 (34)	0.02
BMI (kg/m^2^, mean ± SD)	25.9 ± 4.1	24.9 ± 3.6	0.05
Hb (g/dL, mean ± SD)	13.3 ± 1.6	13.6 ± 1.9	0.24
Platelet (10^3^/mm^3^, mean ± SD)	275.8 ± 84.3	257.5 ± 76.6	0.13
AST (IU/L, mean ± SD)	25.8 ± 12.5	31 ± 28.9	0.21
ALT (IU/L, mean ± SD)	40.4 ± 34.4	34.5 ± 40.2	0.32
Total bilirubin (mg/dL, mean ± SD)	0.7 ± 0.4	1.2 ± 3.9	0.42
Total cholesterol (mg/dL, mean ± SD)	169.9 ± 35	167.5 ± 39.7	0.69
TG (mg/dL, mean ± SD) (median/IQR)	102.5 ± 69.7 88/56.5–125	112.3 ± 70.2 95.5/64.8–139	0.43 0.34
LDL (mg/dL, mean ± SD)	102.7 ± 26.9	98.2 ± 32.5	0.43
HDL (mg/dL, mean ± SD)	51.4 ± 16.4	50.9 ± 14.9	0.84
GBEF (%, mean ± SD)	57.6 ± 39.7	39.3 ± 41.9	0.004

SD: standard deviation; *n*: number; DM: diabetes mellitus; HTN: hypertension; GB: gallbladder; BMI: body mass index; Hb: hemoglobin; AST: aspartate aminotransferase; ALT: alanine aminotransferase; TG: triglyceride; IQR: interquartile range; LDL: low-density lipoprotein; HDL: high-density lipoprotein; GBEF: gallbladder ejection fraction.

**Table 6:** Multivariable analysis for clinical risk factors of steatocholecystitis in patients with calculous cholecystitis.

Variables for steatocholecystitis	OR (95% CI)	*P* value
Age	0.97 (0.94–0.99)	0.004
Sex	NS	NS
HTN	NS	NS
BMI	1.10 (0.99–1.22)	0.06
Total cholesterol	NS	NS
LDL	NS	NS
GBEF	1.01 (0.99–1.02)	0.07

OR: odds ratio; CI: confidence interval; NS: not significant; BMI: body mass index; AST: aspartate aminotransferase; LDL: low-density lipoprotein; GBEF: gallbladder ejection fraction.
